# Graphene-Based Wearable Temperature Sensors: A Review

**DOI:** 10.3390/nano13162339

**Published:** 2023-08-14

**Authors:** Jiajia Liu, Ying Wang, Xiangyang Li, Jiaqi Wang, Yang Zhao

**Affiliations:** Key Laboratory of Cluster Science, Ministry of Education of China, Key Laboratory of Photoelectronic/Electrophotonic Conversion Materials, School of Chemistry and Chemical Engineering, Beijing Institute of Technology, Beijing 100081, China; liujj131295@163.com (J.L.); 17602174372@163.com (X.L.); hhxx1995@126.com (J.W.)

**Keywords:** graphene, wearable temperature sensors, intelligent temperature sensing

## Abstract

Flexible sensing electronics have received extensive attention for their potential applications in wearable human health monitoring and care systems. Given that the normal physiological activities of the human body are primarily based on a relatively constant body temperature, real-time monitoring of body surface temperature using temperature sensors is one of the most intuitive and effective methods to understand physical conditions. With its outstanding electrical, mechanical, and thermal properties, graphene emerges as a promising candidate for the development of flexible and wearable temperature sensors. In this review, the recent progress of graphene-based wearable temperature sensors is summarized, including material preparation, working principle, performance index, classification, and related applications. Finally, the challenges and future research emphasis in this field are put forward. This review provides important guidance for designing novel and intelligent wearable temperature-sensing systems.

## 1. Introduction

Wearable or attached health monitoring smart systems are considered as portable devices for the next generation of telemedicine [1,2]. These devices can monitor physiological information closely related to physical conditions, such as heart rate, wrist pulse, body temperature, blood pressure, etc. [3,4,5,6,7,8]. Intelligent and flexible sensing electronic components are crucial to ensuring that health monitoring systems track and monitor physiological signals in real time [9,10,11], providing a convenient and non-invasive means for disease diagnosis and health assessment [12,13,14]. Since all life activities of the human body are based on metabolism and relatively constant body temperature, real-time monitoring of body surface temperature is one of the simplest, most intuitive, and most effective ways to understand the body’s conditions [15,16,17]. For example, in patients infected with COVID-19, the most prominent clinical manifestation is fever [18]. Continuous real-time detection of human body temperature can not only effectively screen out infected patients from the population, but also inhibit the spread of the disease to a certain extent [19,20].

Commercially available contact temperature sensors are often rigid, have poor fit with human skin, and are difficult to achieve accurate measurement during human movement [21]. As a traditional non-contact temperature sensor, the infrared thermal imager can monitor the temperature of the human body in motion, but its imaging contrast and resolution are not satisfactory [22]. To realize real-time monitoring of dynamic and spatial temperature changes, it is necessary to develop wearable temperature sensors with flexibility, stretchability, biocompatibility, and high sensitivity. Although some flexible temperature sensors, including thermocouples [23,24], thermal resistors [25,26], and thermal response field-effect transistors [27,28,29], have been reported, they are generally large in size and require complex circuitry, making them unsuitable for integration into large-scale wearable devices.

The most practical and common approach to building wearable temperature sensors is by combining lightweight temperature-sensitive materials directly with stretchable or knitwear substrates. This method effectively reduces the overall thickness and maximizes the flexibility of the sensors.

Currently, a wide variety of temperature-sensitive materials have been reported, such as pure metals, metal nanowires [30,31,32], conductive polymers [33,34], and carbon materials (including carbon nanotubes, carbon black, graphene oxide, graphene, etc.) [35,36,37,38]. Among them, graphene has shown superior compatibility in the development of flexible and wearable sensors due to its excellent electronic mobility, tensile strength, flexibility, transparency, and biocompatibility. In particular, graphene has significant thermal conductivity and unique temperature response characteristics. Its conductivity mainly depends on the internal carrier mobility and carrier concentration. With the increase in temperature, the change in carrier concentration becomes the main factor affecting the conductivity. As the carrier concentration increases, the conductivity will also increase and show semiconductor characteristics. In summary, graphene can be used as an important candidate material for temperature-sensing applications [39,40]. Furthermore, graphene, being modified with rich oxygen-containing functional groups during preparation, is easier to integrate with flexible polymer substrates than metal nanomaterials [41,42,43,44,45]. Considering the increasing importance of graphene-based temperature sensors in the development of advanced wearable devices, there is an urgent need for a comprehensive and systematic overview to guide further advancements in this field.

**Figure 1:**
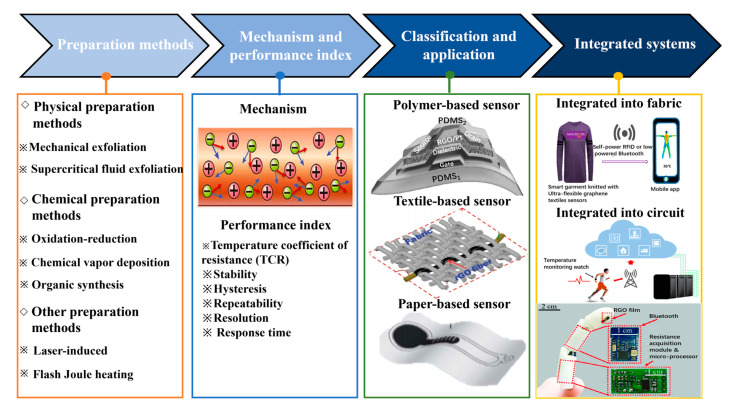
Schematic diagram of the frame of graphene-based temperature sensors. Reprinted with permission from Ref. [46]. Copyright 2023, Microelectronic Engineering. Reprinted with permission from Ref. [47]. Copyright 2016, Advanced Materials. Reprinted with permission from Ref. [48]. Copyright 2018, Advanced Healthcare Materials. Reprinted with permission from Ref. [49]. Copyright 2020, Rsc Advances. Reprinted with permission from Ref. [50]. Copyright 2019, Acs Nano. Reprinted with permission from Ref. [51]. Copyright 2022, Acs Nano. Reprinted with permission from Ref. [52]. Copyright 2019, IEEE Sensors.

In this report, we focus on the research progress of graphene-based wearable temperature sensors and systematically summarize the material preparation methods, the working mechanism, and the performance parameters of the sensors. After that, graphene-based wearable temperature sensors and their multifunctional integrated devices are also clearly classified and reviewed according to the flexible substrate types, as shown in Figure 1. Finally, the current challenges of the sensors are discussed, and future research directions are prospected.

## 2. Graphene Preparation Methods

With excellent properties in force, heat, light, and electricity, graphene possesses great potential for developing a new generation of temperature sensors [53,54,55,56,57]. In order to obtain graphene-based temperature-sensing systems with ideal performance, high-quality graphene needs to be prepared by appropriate methods. At present, the commonly used methods for preparing graphene are roughly divided into physical and chemical preparation.

### 2.1. Physical Preparation Methods

#### 2.1.1. Mechanical Exfoliation

Mechanical exfoliation is a technique used to obtain single or few layers of graphene materials using the friction motion between objects and graphite [58,59]. In 2004, Williams et al. [60] first prepared a small amount of monolayer graphene by repeatedly pasting graphite with tape. Although this method can prepare micron-scale graphene materials, it cannot be industrialized for large-scale mass production due to low production efficiency and low controllability [61].

#### 2.1.2. Supercritical Fluid Exfoliation

**Figure 2:**
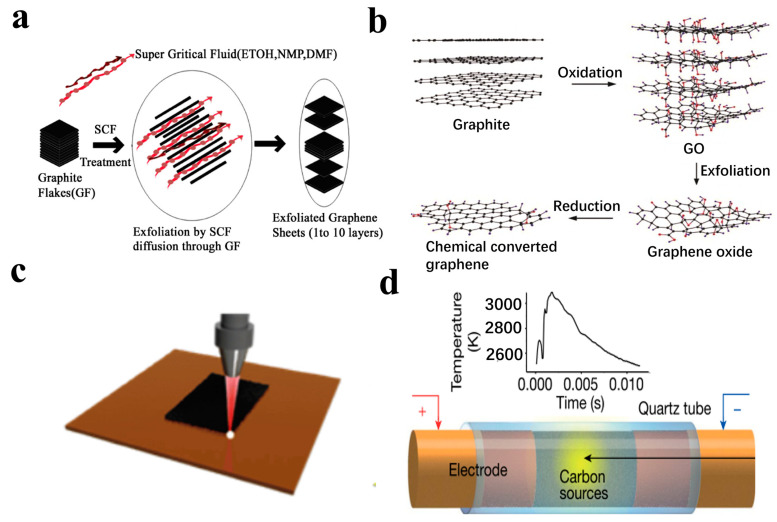
(**a**) Preparation of graphene by supercritical fluid exfoliation [62,63]. (**b**) Preparation of graphene by Hummers’ method [67]. (**c**) Laser-induced graphene [68,69]. (**d**) Preparation of graphene by flash Joule heating [70].

This method utilizes the high dispersion and strong permeability of supercritical fluid to strip graphite [62,63] (Figure 2a). Pu et al. first used this method to prepare graphene with a thickness of 3.8 nm [64]. After that, in order to increase the yield and quality of graphene, some auxiliary means can be used. For example, ultrasonic assistance can be used to obtain a few layers of graphene by its cavitation effect [65], and pyrene-based polymer assistance allows adsorption on the surface of graphite through π–π bond interaction to prepare a uniform and stable dispersion [66].

### 2.2. Chemical Preparation Methods

#### 2.2.1. Oxidation-Reduction

In another method, the crystal structure of graphite is disrupted using strong acids and oxidants, and then reduced to graphene using reducing agents. The most well-known method is the Hummer method [67], which is commonly used by researchers for preparing graphene (Figure 2b). In this method, graphite is first oxidized to graphene oxide (GO) using a combination of H_2_SO_4_, NaNO_3_, and KMnO_4_. The GO solution is then mixed with hydrazine hydrate and ammonia as reducing agents and subjected to ultrasonic dispersion to obtain graphene.

#### 2.2.2. Chemical Vapor Deposition

Chemical vapor deposition (CVD) [71] refers to the decomposition of carbon-containing compounds at high temperatures into carbon atoms, which are deposited on the surface of metals or other substrates to obtain graphene. In 2006, Somaniet al. [72] deposited graphene on the surface of nickel (Ni) at 180 °C using camphor in an argon (Ar) atmosphere by thermochemical vapor deposition. The CVD method allows for the production of graphene with a complete structure and large size, and the number of graphene layers can be controlled by adjusting the growth time, growth temperature, and other conditions, and the controllability is strong. However, the complicated preparation process and high production cost limit the widespread application of this method [73].

#### 2.2.3. Organic Synthesis

The organic synthesis method involves the conversion of small molecular aromatic compounds into graphene nanoribbons, which are then further treated through dehydrogenation to obtain graphene [74]. The method yields graphene with high quality, but it is associated with challenges such as high preparation cost, low efficiency, and potential environmental pollution.

### 2.3. Other Preparation Methods

#### 2.3.1. Laser Induction

Laser-induced graphene (LIG) is a three-dimensional porous material that is generated when a laser beam is irradiated on certain carbon precursors [68,69] (Figure 2c). In 2014, Tour et al. [75] discovered that a CO_2_ infrared laser could produce LIG with a three-dimensional porous structure on the surface of polyimide (PI). LIG exhibits high porosity, excellent electrical conductivity, and good mechanical flexibility. It is possible to directly create pre-designed LIG patterns that can be directly fabricated on various carbon materials such as polyimide, wood, lignin films, polysulfone, cross-linked polystyrene, and Teflon polysulfone. The microstructure, conductivity, chemical composition, and heteroatom doping are controllable. This selective and cost-effective patterning technology minimizes the use of raw materials and reduces environmental impact.

#### 2.3.2. Flash Joule Heating

In 2020, Tour et al. [70] introduced a flash Joule heat method for synthesizing graphene. This innovative process enables the conversion of various carbon-containing precursors into high-quality graphene. It has the capability to produce large quantities of valuable graphene sheets from almost any carbon source. Moreover, the preparation process is rapid, with only 1 s required to produce 1 g of graphene.

## 3. Thermal Response Mechanism and Performance Index of Graphene-Based Temperature Sensors

### 3.1. Thermal Response Mechanism

Graphene-based temperature sensors are considered one of the most promising new-generation temperature sensors. The unique characteristics and structure of graphene make it suitable for the construction of temperature sensors. For example, ultra-high thermal conductivity makes graphene-based temperature sensors have an ultra-fast thermal response, the huge specific surface area makes it have a much larger thermal contact area than other materials, and the ultra-high carrier mobility also fully ensures that the temperature sensor can respond quickly to temperature changes. These characteristics have attracted significant attention from scholars who are studying and discussing the temperature-sensitive mechanism of graphene-based temperature sensors.

**Figure 3:**
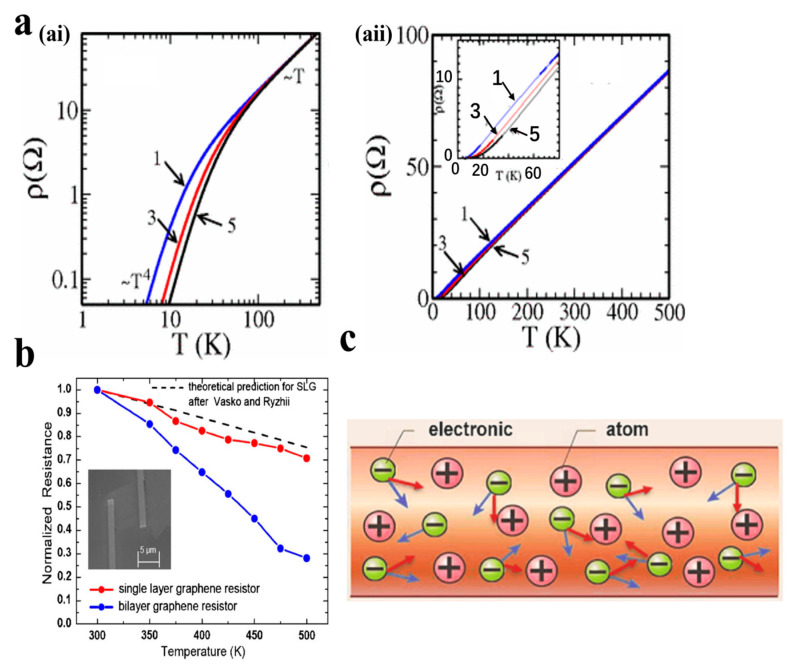
(**a**) Temperature dependence of the resistivity [76]: (**ai**) at low temperature; (**aii**) at high temperature. (**b**) Temperature test of single- and double-layer graphene [46]. (**c**) Thermal response mechanism diagram of graphene [77].

Hwang et al. [76] calculated the transport properties of graphene in the temperature range of 500 K by considering the relationship between temperature and phonon scattering of carrier density (Figure 3a). The study confirmed that the changes in the electrical properties of graphene are related to electron-phonon scattering. After that, Shao et al. [46] carried out temperature tests on graphene prepared by mechanical exfoliation. They observed that as the temperature increased from 300 K to 500 K, the resistance of monolayer and bilayer graphene decreased by 30% and 70%, respectively (Figure 3b). At the same time, they also found that the temperature characteristics of graphene should not only consider electron-phonon scattering, but also consider the composite scattering of electrons and other charged particles.

In summary, the temperature response of graphene involves both the scattering effect of electron-phonons and the scattering effect of other charged particles at high temperatures. When temperature is applied to graphene, the electron motion direction is altered due to the scattering of electrons and phonons, as well as the combined scattering of electrons and other charged particles. This results in a change in resistivity [77] (Figure 3c). It is important to note that this understanding of the temperature response of graphene does not consider the influence of defects and substrates.

### 3.2. Performance Indexes

To evaluate whether a temperature sensor has a stable health monitoring capability, it is necessary to start with the following key indicators: sensitivity, stability, resolution, repeatability, and response time. The sensitivity of the temperature sensor is determined by the resistance temperature coefficient [78,79,80]. The general equation of the temperature change of a thermistor is [16]:

(1) $$R_{t}=R_{0}exp\beta (\frac{1}{T}-\frac{1}{T_{0}})$$

where *R_t_* is the resistance value at temperature *T*, *R*_0_ is the resistance value at *T*_0_ (reference temperature), and *β* is the material coefficient of the thermistor. Taking the logarithm of both sides of the above equation, we can obtain the linear relation between ln (*R_t_*) and 1/*T*, where *β* represents the slope of the output curve (*K*). The temperature coefficient *α* of the thermistor is:

(2) $$\alpha =\frac{1}{R_{t}}\frac{{dR}_{t}}{dT}=-\frac{\beta }{T^{2}}$$

where *α* is the percentage of resistance value change with unit temperature change, %/*K*. The resistance temperature coefficient [81] is divided into the positive temperature coefficient (PTC) [82] and the negative temperature coefficient (NTC). When the temperature coefficient is positive, the resistance value of the sensors will increase with the increase in temperature. When the temperature coefficient is negative, the resistance value of the sensors will decrease with the increase in temperature.

The stability evaluation of the temperature sensor is achieved by recording and analyzing the resistance fluctuation of the sensor multiple times at the same time interval at a constant temperature. In the case of small temperature changes, the temperature sensor needs to be very sensitive and stable to detect such small changes [83]. If the resistance value of the sensor itself changes greatly, it will interfere with the temperature detection. Therefore, the stability of the sensor is fundamental in detecting small temperature changes. Generally speaking, the condition for judging the stability of the device is that the relative change rate of the resistance does not exceed 1% [84].

To further judge the performance of temperature sensors, their hysteresis [85] and repeatability [86] also need to be known. Hysteresis is to point in the process of heating and cooling at the same temperature corresponding to the biggest difference in the resistance, the availability of the maximum error of the hysteresis

(3) $$\;y_{H}=\frac{{(▲H}_{max})}{y_{fs}}\times 100\%$$

where$\;▲H_{max}$ represents the maximum change value in the process, and $y_{fs}$ represents the full-scale output value.

Reproducibility is one of the most important properties of flexible temperature sensors, which affects the reuse of temperature sensors in daily life. With the increase in the number of rising and cooling cycles, the activation energy on the surface of graphene sheets will also change, a more stable network structure will be established between layers, and the electrical conductivity will become more stable. Therefore, graphene has good temperature-sensitive repeatability. The non-repeating index is defined as follows [87]:

(4) $$e_{z}=\frac{{▲}_{max}}{y_{fs}}\times 100\%$$

where ${▲}_{max}$ represents the maximum non-repeating error of the output, and $y_{fs}$ represents the full-scale output. 

In practical applications, resolution and response time [88] are also important performance indexes of temperature sensors. Resolution refers to the sensor’s ability to detect subtle changes in temperature. A high-resolution sensor can detect even small temperature variations. Response time, on the other hand, refers to the time it takes for the sensor to respond to a change in ambient temperature. A smaller response time means the sensor can quickly react to temperature changes. Both higher resolution and smaller response time are beneficial for the detection performance of temperature sensors.

Furthermore, to assess the flexibility of the sensors, a bending test is conducted. This test involves subjecting the sensor to multiple bending cycles to evaluate its temperature detection capability. It is observed that the temperature detectability of the device remains normal even after bending. This can be attributed to the tunneling effect generated by the graphene film during the bending process. This effect helps offset the deformation caused by partial bending and the shrinkage of the carbon-carbon bond spacing. As a result, the conductive properties of graphene remain largely unchanged during the bending process.

## 4. Classification and Application

Wearable temperature sensors primarily utilize the change in the electrical signal caused by the temperature change of thermal sensitive materials to realize real-time temperature monitoring. To ensure accurate measurement of human temperature, in addition to the reliable sensitivity of the sensing materials, these sensors need to be accompanied by flexible substrates that can conform closely to the human skin. Flexible substrates are characterized by uniform deformation, high elasticity, and the ability to be easily processed into portable forms such as scrolls. In addition, the substrates should possess a low modulus of elasticity, making them resistant to damage under folding and bending, thereby meeting the requirements of mechanical properties for flexible devices. There are various substrate materials available with soft properties, such as flexible thermoplastic polymers (polycarbonate, polyimide, polyurethane, etc.), thermosetting polymers (polydimethylsiloxane), textiles, and paper. Different types of soft substrates can be chosen based on the specific application scenarios. 

### 4.1. Based on Polymer Substrates

Polymer materials are commonly used as flexible substrates due to their outstanding flexibility, low cost, and ability to be easily rolled into structures. Some of the polymer materials used as flexible substrates include polyimide (PI) [89], polyethersulfone (PES), polyether imide (PEI) [90], polyethylene naphthalate (PEN), and polyethylene terephthalate (PET) [91].

**Figure 4:**
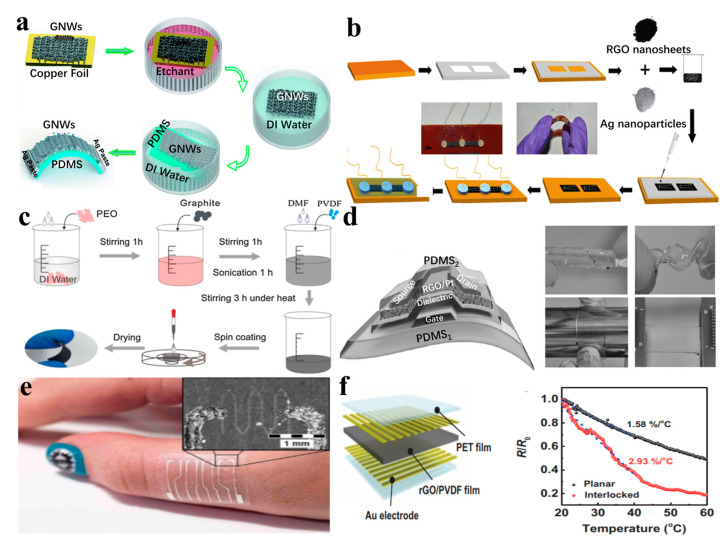
(**a**) The preparation process of wearable temperature sensors based on GNW/PDMS [92]. (**b**) Fabrication process of flexible temperature sensor based on rGO and Ag nanoparticles [93]. (**c**) Preparation of multimodal strain and temperature sensors [47]. (**d**) Structure and morphology of transparent stretchable temperature sensor [94]. (**e**) Photograph of the sample, with sensor array of four sensors, being attached to the skin [95]. (**f**) A flexible temperature sensor composed of polyvinylidene fluoride (PVDF) and rGO was designed to imitate the fingerprint structure and epidermal microjunction structure of the human fingertip [96].

For example, Yang et al. [92] reported a simple wearable temperature sensor using graphene nanowalls (GNWs) embedded in polydimethylsiloxane (PDMS) as the substrate (Figure 4a). This sensor exhibits an exceptionally high thermal response, surpassing traditional temperature sensors. It has a temperature coefficient of resistance (TCR) of 0.214%/°C, which is three times higher than that of conventional similar products. The PDMS substrate, known for its flexibility and high coefficient of thermal expansion (TCE), enables the stretchability of GNWs. During heating, the PDMS substrate expands radially, forming long conductive channels between the GNWs. Upon cooling, the conductive channel returns to the original state with fast response, considerable recovery speed, and high stability, which can monitor the temperature in real time. Similarly, Neella et al. [93] prepared a resistive temperature sensor by coating a solution mixed with reduced graphene oxide (rGO) and Ag nanoparticles onto a PI substrate. The experimental results show that the sensor demonstrates excellent temperature-sensing characteristics with a temperature coefficient of resistance (TCR) of −1.64 × 10^−3^ Ω K^−1^. It also exhibits a fast measurement response time of 470 ms, which is much faster than most commercial temperature sensors. Additionally, the sensor displays good repeatability and stability (Figure 4b).

Some polymer materials, in addition to their good tensile ductility, also have biocompatibility, making them suitable for human body temperature monitoring. For instance, polyurethane (PU) is commonly used in this context. Hang et al. [47] developed a temperature sensor by preparing a hybrid aerogel of PEDOT: PSS and rGO and injecting it onto a PDMS substrate (Figure 4c). The resulting sensor exhibits multimodal strain capability, high resolution, and repeatability in the temperature range of 34.0 °C to 42.0 °C. The resistance of the sensor decreases with increasing temperature, with a TCR of 1.69%/°C. Based on the high transparency of most polymer substrates, it is possible to develop fully transparent sensors to meet specific application requirements. Trung et al. [94] developed a transparent and stretchable temperature sensor that can be easily attached to objects or human skin (Figure 4d). Conductive and temperature-sensitive rGO nanosheets were inserted into an elastic PU matrix to form a composite material as a temperature-sensing layer. This sensor can withstand tensile strains of up to 70% and exhibits a sensitivity of TCR = 1.34%/°C, capable of detecting temperature changes as small as 0.2 °C. Furthermore, Vuorinen et al. [95] utilized inkjet printing of graphene/PEDOT: PSS inks and screen-printing of silver inks to prepare sensing layers, which were then printed on a PU plaster. The resulting sensor operates within a temperature range of 35–45 °C, with a TCR of −0.06%/°C (Figure 4e).

Incorporating bionic structures into the design of flexible sensors can effectively improve their compatibility with wearable devices. To this end, Park et al. [96] designed a flexible temperature sensor by imitating the fingerprint structure of the human fingertip and the epidermodermal micronodular structures. The sensor is composed of polyvinylidene fluoride (PVDF) and rGO. This bionic design enables the sensor to sensitively detect temperature changes, exhibiting a temperature coefficient of resistance (TCR) of up to 2.93%/°C (Figure 4f). This approach leverages nature-inspired structures to improve the performance and compatibility of flexible sensors in wearable applications.

### 4.2. Based on Textile Substrates

Textiles are lightweight, flexible, deformable, and breathable, and ensure comfort for the human body. As a result, they have great potential in the manufacturing of flexible electronics for personal health management. Graphene can be incorporated into textile products through various methods [97,98,99], with the following two being the most commonly used. The first method is the functional modification of fabrics or fibers by the chemical or physical coating of carbon materials such as graphene, graphene oxide, and reduced graphene oxide. This method is simple, easy to apply, scalable, and has received high praise from scholars and industry experts. The second method is the chemical vapor deposition of graphene on metal mesh templates, followed by removal of the templates through acid treatment. This process results in the formation of a graphene fabric structure, commonly known as graphene fabric.

**Figure 5:**
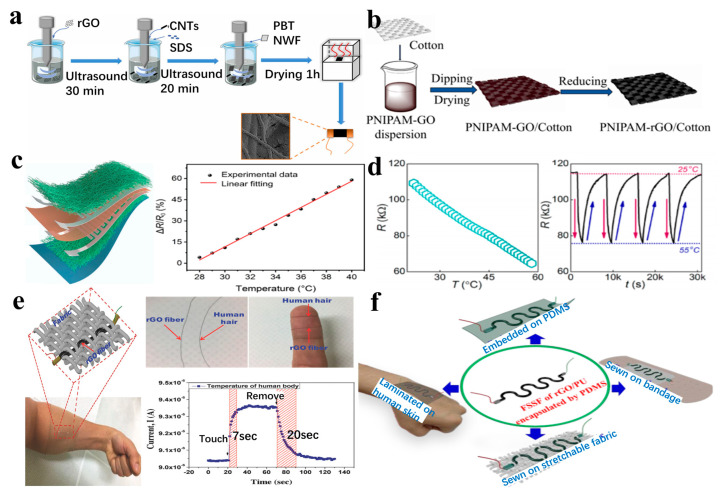
(**a**) A wearable temperature sensor is prepared by combining non-woven fabric with rGO/CNT [100]. (**b**) The process of loading PNIPAM-rGO on cotton fabric [101]. (**c**) Waterproof breathable cotton fabric composite decorated with rGO and CNTs [50]. (**d**) The morphology of the fabric and its response to heat [48]. (**e**) Wearable temperature sensor based on single microfiber [102]. (**f**) A strain-insensitive stretchable temperature sensor for rGO/PU composites was prepared by the fiber-spinning method [103].

Wang et al. [100] developed a wearable temperature sensor by ultrasonically combining polybutylene terephthalate melt-blown non-woven fabric (PBTNW) with rGO and carbon nanotubes (CNTs) (Figure 5a). The test results showed that the sensor revealed high sensitivity (−0.737% °C^−1^), with a resolution of 0.1 °C within the temperature range of 25 °C to 45 °C. Tan et al. [101] achieved a temperature-sensitive effect on cotton fabric by applying multiple dipping and drying cycles of PNIPAM-GO, followed by reduction with hydrazine hydrate. The resistance value of the fabric exhibited reversibility with temperature changes in the range of 25–50 °C (Figure 5b). Yin et al. [50] prepared a waterproof breathable cotton fabric composite decorated with rGO and CNTs by solution osmosis. The composite material served as a sensing layer capable of highly sensitive detection of pressure and temperature stimuli. The device demonstrated a good linear response in the temperature range of 28~40 °C (Figure 5c) and successfully applied to non-contact real-time monitoring of human respiratory signals.

In addition to functionalizing textile fabrics, graphene can also be loaded onto a single woven yarn or microfiber, which can be easily integrated into clothing. Afroj et al. [48] developed a graphene-based wearable textile that is flexible, washable, and bendable, operating within the temperature range of 25–55 °C (Figure 5d). Graphene-based inks were used to dye (coat) textile yarns using high-speed yarn dyeing techniques. The resulting graphene textile sensor exhibited excellent temperature sensitivity, washability, and extremely high flexibility. Similarly, Trung et al. [102] reported a wearable temperature sensor based on a single microfiber for the first time (Figure 5e). Graphene fibers were prepared by wet spinning and in situ reduction processes. GO gel fibers were reduced in a hydrogen iodide/acetic acid solution with a volume ratio of 1:20, followed by thermal treatment with deionized water in an autoclave at 200 °C for 24 h. This process produced a single RGO fiber with a diameter of approximately 40 μm, close to that of human hair. The device operated within the temperature range of 30–80 °C, exhibited a fast response time (7 s), good recovery time (20 s), and maintained its response even when subjected to bending radii up to 4 mm and cycling bends up to 10,000 times. In the same year, Trung et al. [103] also prepared a strain-insensitive stretchable temperature sensor based on rGO/PU composites using a simple fiber-spinning method, which showed higher thermal responsiveness and remarkable mechanical deformation. The sensitivity of the temperature sensor was 0.8%/°C, the stretchability was up to 90%, and the sensing resolution value is 0.1 °C (Figure 5f). The sensors were sewn onto stretchable bandages and attached to the human body to continuously and steadily detect changes in skin temperature during various body movements.

### 4.3. Based on Paper Substrates

To address the issue of electronic waste, researchers are focusing on developing paper substrates for sensors. Paper, being hydrophilic and capillary, can be easily modified through simple methods such as soaking, coating, spraying, or printing. Paper-based sensors offer several advantages including low cost, recyclability, and environmental degradation. These paper-based sensors are widely utilized in various applications such as detecting pressure [104], temperature, humidity, and more. The versatility and accessibility of paper make it an ideal substrate for developing sensors that are both environmentally friendly and cost-effective. 

**Figure 6:**
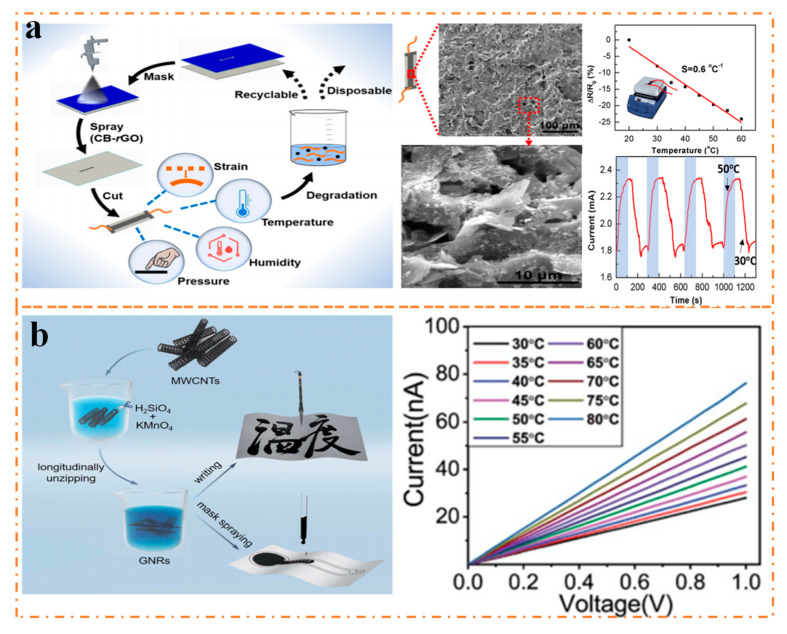
(**a**) The preparation process and performance display of paper multimode sensor [49]. (**b**) Preparation process and performance of disposable flexible temperature sensor [105].

Liu et al. [49] developed a flexible paper-based multimodal sensor that could simultaneously detect strain, humidity, temperature, and pressure with a single device. They achieved this by spraying a moisture dispersion of carbon black (CB) and rGO onto the paper, repeating the spray-drying process multiple times to form a sensitive layer. The micromorphology of this sensitive layer is shown in Figure 6a, where CB particles are adsorbed on the surface of the rGO and then arranged hierarchically. The device operates in the temperature range of 20–60 °C and has a sensitivity of 0.6%/°C, higher than commercial Pt sensors. Besides, since the paper is made from plant fibers, the sensor easily degrades in water but can be reused after undergoing the soil-drying process, making it a reliable option.

Likewise, Gong [105] reports on a disposable flexible temperature sensor that uses synthetic graphene nanoribbon (GNR) ink with high thermal sensitivity, which can be written or sprayed onto commonly used paper (Figure 6b). The sensor serves as an economical and practical disposable health monitoring equipment, employing inexpensive materials and simple technology. At high temperatures, the GNR forms appropriate band gaps and local traps within the forbidden band, thus enhancing thermal activation transmission and resulting in high thermal sensitivity. Notably, the sensor operates within a temperature range of 30–60 °C, exhibiting a high sensitivity of 172%, fast response time of 0.5 s, high-temperature resolution of 0.2 °C, and flexibility.

## 5. Wireless Sensor Network

### 5.1. The Composition and Working Mechanism of Wireless Sensor Network

**Figure 7:**
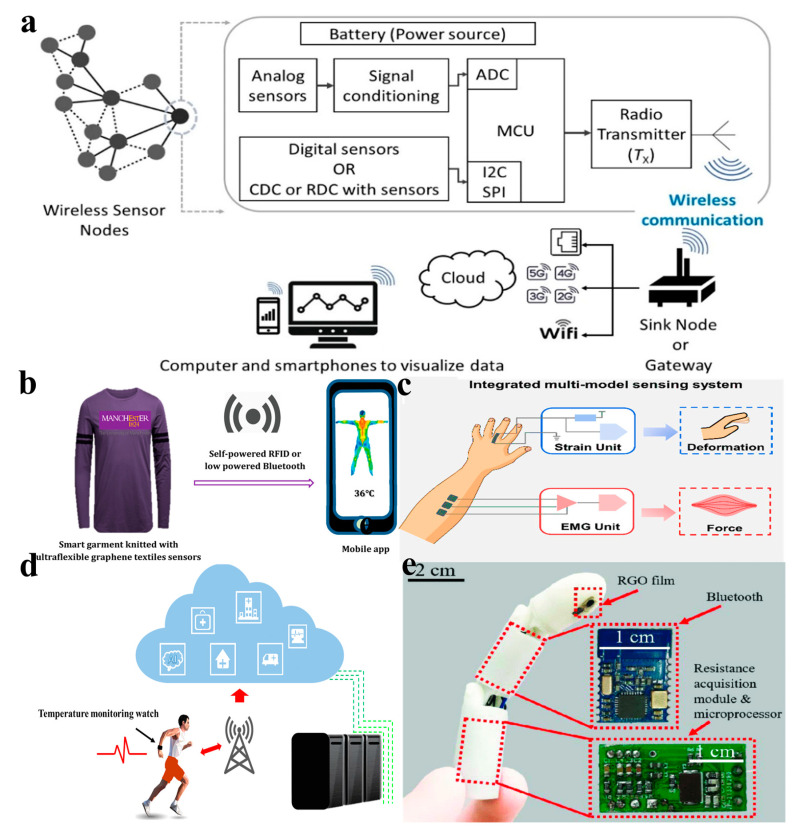
Wireless sensor networks and applications. (**a**) Schematic diagram of a wireless sensor network [22]. (**b**) Intelligent clothing application diagram [48]. (**c**) Washable electronic textiles with multiple functions [51]. (**d**) PANI/graphene-based temperature sensor integration [52]. (**e**) A flexible rGO temperature sensor on a 3D-printed robot fingertip [108].

A wireless sensor network (WSN) is composed of tens of thousands of sensor nodes that are interconnected through wireless communication technology. Unlike a traditional local area network, WSN offers stronger communication stability and ensures the freshness, integrity, and confidentiality of data during transmission. The sensor nodes in a WSN comprise several units, including the data acquisition unit, data transmission unit, data processing unit, and energy supply unit. These units work together to collect data from the surrounding environment, transmit it wirelessly, process it if necessary, and ensure a continuous supply of energy to the sensor node. Wireless sensor networks are key technologies for continuous sensing and wireless transmission of data, especially for the implementation of the Internet of Things (IoT) [106,107]. Figure 7a represents a schematic diagram illustrating the structure and connectivity of a wireless sensor network [22].

Typically, sensors provide data output in the form of digital or analog signals. In the dynamic detection of human physiological conditions, the capacitance or resistance of the sensors will change with the change of sensor parameters. At this point, a digital converter (CDC) or electro–digital conversion (RDC) is required to convert the sensor’s capacitance or resistance data into digital form. However, CDC or RDC has limited ability to process and compute data. Therefore, additional units (such as microcontroller units (MCUS)) that can perform data processing are required. The processed data is then transmitted wirelessly via a radio transceiver to a receiver node or gateway/base station. In addition, the receiver node or gateway forwards this data to the cloud over Wi-Fi, Ethernet, or cellular networks (e.g., 2G to 5G).

### 5.2. Applications of Wireless Sensor Network

Wearable temperature sensors have remarkable properties such as high tensile strength, skin compatibility, and high sensitivity and resolution, which can be combined with wireless sensor networks to realize the remote dynamic monitoring of human health conditions. Continuous dynamic monitoring of body temperature requires continuous access and manipulation of sensory data. Cloud computing technology advancements offer an excellent opportunity to further analyze and store sensor data in the IoT cloud. This enables users to easily retrieve data from any Internet-connected device on demand. Different technologies can be used to achieve wireless transmission, such as Bluetooth low power, radio frequency identification (RFID), near-field communication (NFC), ZigBee, and long-range wide-area network. To achieve remote dynamic detection of physiological signals, several factors need to be considered when selecting the communication architecture. These factors include the cost of sensing equipment, the number of sensor nodes, the target communication range, equipment lifespan, and power consumption. In terms of power consumption, graphene-based temperature sensors are commonly employed due to their low power consumption, generally ranging from tens to hundreds of microwatt (μW) [107,109,110,111].

Integrated fabric or fiber-based temperature sensors have a wide range of applications due to the flexibility and wearability of the fabric itself, as well as the washable properties of graphene-based fabrics. Afroj et al. [48] demonstrated a smart garment concept that integrates knitted temperature sensors, other wireless sensors (such as but not limited to strain, pressure, and humidity), and RFID technology. The temperature data collected by the sensors can be sent to a mobile application through an NFC reader. Additionally, the garment can connect to low-power Bluetooth devices to transfer data to other devices (as shown in Figure 7b). Zhang et al. [51] prepared a graphene-modified e-textile with hydrophilic, breathable, biocompatible, and washable properties. The fabric was prepared with sericin assistance, enabling a comfortable multisensor-integrated textile. The fabric’s knitted structure is well-preserved with a conformal coating of hydrophilic sericin-graphene sheets, providing excellent electrical conductivity, hydrophilicity, biocompatibility, air permeability, and flexibility. These properties ensure both electronic functionality and wearing comfort. Building upon this foundation, an integrated multisensor textile was developed, which could collect and analyze electromyographic and mechanical signals at the same time, realizing the recognition and differentiation of complex human movement (Figure 7c).

In addition to integrated fabrics, portable integrated sensors are widely used for real-time temperature monitoring. These sensors can be attached to the wrist and worn as a bracelet, allowing for convenient monitoring in various situations. Ren et al. [52] designed a temperature sensor based on graphene/PANI with high sensitivity (1.60%/°C), resolution (0.3 °C), and short time response (0.7 s) within the temperature-sensing range of 25~40 °C. Based on this, the temperature sensor can be combined with a readout circuit and a filter circuit to create an integrated device, such as a sports bracelet. This device can be used to monitor the athlete‘s body temperature in real time during running and upload the health data to the cloud for remote intelligent medical treatment (Figure 7d). In dangerous conditions, sensors can be integrated into certain parts of a robot for remote monitoring. Zhou et al. [108] studied a new type of robotic finger with an rGO-based flexible temperature sensor, an integrated circuit, and an external Bluetooth for real-time wireless data transfer to the phone (Figure 7e). The temperature-sensing performance of the sensor was measured in the range of 303~353 K, and the resistance temperature coefficient of −1.223%/K was obtained.

These portable integrated sensors offer the advantage of real-time temperature monitoring in different scenarios, whether for personal health monitoring during physical activities or for remote monitoring in hazardous environments. The data collected by these sensors can be transmitted wirelessly for further analysis and decision-making.

## 6. Conclusions and Challenges

Compared to commercially available rigid temperature sensors, wearable temperature sensors developed using graphene materials display outstanding flexibility and shape adaptability. These sensors can be closely fitted to various parts of the human body, making them highly suitable for physiological monitoring and disease prediction. This paper gives a comprehensive overview of recent achievements in graphene-based wearable temperature sensors, including the preparation methods of graphene, the performance index, and the response mechanism of the sensors. Then, the temperature sensors are classified according to the types of flexible substrates, and the corresponding application scenarios are further introduced. Through the summary of relevant application developments, it is evident that reliable intelligent wearable temperature-sensing systems require the collaboration of multicomponents. This necessitates efficient integration of the sensing unit, power supply unit, communication module, and data processing module. This integration presents a major research trend in this field, as it sets higher requirements for achieving seamless cooperation among these components. In conclusion, graphene-based wearable temperature sensors offer significant advantages over rigid sensors, and they have great potential in various applications related to physiological monitoring and disease prediction.

Graphene-based wearable temperature sensors have good application prospects because they are suitable for the integration of textiles or skin, can be closely attached to various parts of the human body, and ensure the accuracy of real-time monitoring results. At present, a lot of research has been carried out on the use of graphene to manufacture wearable temperature sensors. The main performance is that there are more and more methods for preparing high-quality graphene. Therefore, the performance of the sensor can be regulated to a certain extent by different preparation methods. Due to the limitations of graphene as a heat-sensitive material, the most suitable temperature range of graphene-based temperature sensors is 25–55 °C, and the minimum resolution can reach 0.2 °C. It not only covers the temperature range of human health monitoring, but also can accurately measure the parts with a small temperature difference. In addition, based on the portability of wearable sensors, sensors are combined with wireless sensor networks to achieve remote dynamic monitoring of human health status and receive data information in real time.

Graphene-based wearable temperature sensors have good application prospects because they are suitable for the integration of textiles or skin, can be closely attached to various parts of the human body, and ensure the accuracy of real-time monitoring results.

Although graphene has been used in a variety of flexible strain [112,113], pressure [114], temperature [92,115,116], humidity [117,118], and multimodal sensors [119,120], many practical applications are still challenging. These challenges include the following:

(1) The complex and high-cost preparation process of graphene-based temperature sensors hinders their wide application. To overcome this limitation, there is an urgent need to develop more low-cost and simple preparation methods. Researchers should focus on finding alternative techniques that can streamline the production process and reduce the overall cost of manufacturing these sensors. This will enable their widespread adoption in various fields.
(2) One of the challenges faced by graphene-based temperature sensors is the crosstalk between different stimulation signals. This can make it difficult for the sensor to accurately distinguish the signal to be monitored from other signals, affecting its performance and reliability. Addressing this challenge requires the development of advanced signal processing algorithms and techniques that can effectively filter out unwanted interference and isolate the desired temperature signal. Researchers should continue to explore innovative approaches to enhance the signal-to-noise ratio and improve the specificity of these sensors.
(3) While high performance is essential, the comfort of wearable temperature sensors is also crucial for their long-term use. Some substrate materials used in these sensors may have poor air permeability, making them uncomfortable to wear for extended periods. To address this issue, it is important to explore alternative substrate materials that offer better breathability and comfort. Additionally, the design and fabrication of the sensors should prioritize user comfort, ensuring that they are lightweight, flexible, and non-irritating to the skin. This will enhance user acceptance and enable the sensors to be worn for longer durations without discomfort.

Indeed, with the advancement of simple preparation methods for graphene materials, the field of flexible graphene-based sensors is expected to make significant progress in the future. These sensors will benefit from the high selectivity and comfort they offer.

Simplifying the preparation process for graphene materials will make them more accessible and cost-effective, enabling wider adoption of flexible graphene-based sensors. Researchers are actively exploring various techniques to simplify the fabrication process, such as solution processing, printing, and scalable manufacturing methods. These advancements will contribute to the mass production of graphene-based sensors, making them more readily available for various applications.

Furthermore, the high selectivity of graphene-based sensors allows for precise and accurate detection of specific signals or stimuli. This selectivity can be enhanced by functionalizing the graphene surface or integrating additional sensing elements. The ability to selectively detect and monitor specific parameters will enable these sensors to be applied in diverse fields, including healthcare, environmental monitoring, and wearable technology.

Comfort is another crucial aspect to consider in the development of wearable sensors. The flexibility and lightweight nature of graphene-based sensors make them well-suited for comfortable and prolonged use. Researchers are continually exploring new substrate materials and designs that prioritize user comfort, breathability, and skin compatibility. These efforts will ensure that graphene-based sensors can be comfortably worn for extended periods without causing any discomfort or irritation.

Overall, the combination of simple preparation methods for graphene materials, high selectivity, and comfort in flexible graphene-based sensors holds great promise for future advancements in sensor technology. These sensors are expected to find widespread applications in various industries, contributing to advancements in healthcare, environmental monitoring, and wearable technology.

## Data Availability

Not applicable.
